# Immunological and metabolic profiles associated with active *Giardia intestinalis* infection in adults with metabolic disorders: an exploratory analysis

**DOI:** 10.3389/fimmu.2026.1936690

**Published:** 2026-10-27

**Authors:** Sylwia Klimczak, Kacper Packi, Alicja Rudek, Sylwia Wenclewska, Marcin Kurowski, Agnieszka Śliwińska

**Affiliations:** 1 Department of Nucleic Acid Biochemistry, Medical University of Lodz, Lodz, Poland; 2 AllerGen Center of Personalized Medicine, Piotrkow Trybunalski, Poland; 3 Department of Immunoparasitology and Molecular Allergology, Jan Dlugosz University, Czestochowa, Poland; 4 Diabetology and Internal Medicine Department, Provincial Hospital in Sieradz, Sieradz, Poland; 5 Department of Immunology and Allergy, Medical University of Lodz, Lodz, Poland

**Keywords:** cytokines, exploratory analysis, *Giardia intestinalis*, immunometabolic profiling, metabolic disorders

## Abstract

**Background:**

Giardiasis may present with nonspecific gastrointestinal symptoms, and conventional diagnostic approaches may not fully capture host-response differences in clinically complex patients. This study characterized immunological and metabolic measurements associated with active *G. intestinalis* infection in adults with metabolic disorders and examined their differences according to infection status and treatment stage. An exploratory secondary analysis assessed whether selected variables could jointly discriminate infection status.

**Methods:**

In this prospective observational study, 84 adults with metabolic disorders aged 40–65 years were included at baseline and classified according to *G. intestinalis* infection status; 80 participants were included in the final analytical sample. Anti-*Giardia* IgM and IgG, multiplex cytokines, chemokines and growth factors, anthropometric measures, and metabolic parameters were assessed. Differences according to infection status and treatment stage were examined using non-parametric tests, Spearman correlations, and logistic regression. Selected variables were subsequently evaluated using multivariable logistic regression and receiver operating characteristic (ROC) analysis. Internal model performance was assessed using bootstrap resampling and stratified 10-fold cross-validation within the study cohort.

**Results:**

Active *G. intestinalis* infection was associated with lower glucose, BMI, and HOMA-IR values and with differences in selected inflammatory mediators. IgM, IL-12p40, IL-8, RANTES, and IL-2R were among the variables associated with infection status in regression analyses. An exploratory integrated model including glucose, BMI, IgM, RANTES, IL-12p40, and IL-2R showed an apparent AUC of 0.953. Internal performance assessment using bootstrap resampling and stratified 10-fold cross-validation yielded similar estimates within the study cohort. Differences in apparent AUC between the integrated, immunological, and metabolic models were interpreted descriptively because no formal statistical comparison of ROC curves was performed.

**Conclusions:**

In this study population, active *G. intestinalis* infection was associated with distinct immunological and metabolic patterns. The exploratory multivariable analysis identified a combination of immunological and metabolic variables with high apparent discriminatory performance within the present cohort. These findings are hypothesis-generating, and the identified predictor combination requires evaluation in substantially larger independent cohorts with prospective model development and external validation before its predictive or clinical utility can be determined.

## 1 Introduction

*Giardia intestinalis* infection remains a common but frequently underrecognized cause of gastrointestinal dysfunction in adults (1, 2). Although giardiasis is classically associated with diarrhea, malabsorption, and weight loss, its clinical presentation is often heterogeneous and nonspecific, particularly in patients with coexisting chronic conditions (2–4). In routine practice, infected individuals may present with mild or fluctuating symptoms, such as intermittent abdominal discomfort, bloating, dyspepsia, nausea, or altered bowel habits, which may overlap with other gastrointestinal and metabolic disturbances (3, 5). This diagnostic ambiguity increases the risk of delayed recognition, especially in patient groups in whom gastrointestinal complaints may be attributed to pre-existing metabolic disease rather than to parasitic infection (4, 6).

Giardiasis remains an important food- and water-borne parasitic infection in Europe. According to the European Centre for Disease Prevention and Control (ECDC), 10,894 confirmed cases were reported in the EU/EEA in 2022, corresponding to a notification rate of 3.9 cases per 100,000 population (7). Reported rates varied substantially between countries, with the highest notification rates observed in Luxembourg, Belgium, and Spain. The highest age-specific notification rates occurred among children aged 0–4 years, while infections were also reported across adult age groups (7). These surveillance data should be interpreted in the context of differences in national surveillance systems and likely under-ascertainment of infection.

In Poland, giardiasis is a notifiable disease, and surveillance data indicate substantial temporal and regional variation in reported cases. In 2021, 559 cases were reported nationwide, corresponding to an incidence of 1.5 per 100,000 population, with cases reported in all voivodeships and the highest incidence in the Podlaskie Voivodeship (9.1 per 100,000) (8). Children represented the largest age group among reported cases. More recent surveillance data indicate an increase in reported cases, with 1,771 cases recorded in 2023 (4.7 per 100,000 population) (8). These figures represent reported cases rather than population-based prevalence and therefore may underestimate the true burden of infection. Although reported giardiasis is most frequent among young children, infection also occurs in adults, including individuals evaluated for gastrointestinal or metabolic conditions.

Beyond its intestinal manifestations, *G. intestinalis* infection may influence nutritional status, mucosal integrity, and host inflammatory balance (9, 10). These effects may interact with metabolic and immune pathways and are therefore of particular interest in patients with metabolic disorders (10–12). Patients with metabolic disorders may represent a clinically relevant population in which host–pathogen interactions are influenced by chronic low-grade inflammation, altered immune responsiveness, and disturbed metabolic balance (13–18). Although direct epidemiological evidence linking metabolic disorders specifically to increased susceptibility to *G. intestinalis* infection remains limited, experimental and clinical evidence supports the biological plausibility of altered host responses in this population.

Evidence specifically addressing giardiasis in adults with metabolic abnormalities remains limited. In a cross-sectional study of 104 individuals with morbid obesity, *G. intestinalis* was detected in 8.7% of participants, and intestinal parasite colonization was associated with lower HOMA-IR levels, although the study did not establish a causal relationship between parasitic colonization and metabolic status (19).

Similarly, a clinical study including adults with diabetes mellitus reported *G. intestinalis* antigen positivity in 15% of diabetic patients compared with 7% among patients with dyspepsia and no positive results among healthy controls (20). These findings suggest that giardiasis may occur in adults with metabolic disorders, although the available evidence is derived from selected clinical populations and does not establish a general population prevalence or causality.

In individuals with metabolic disorders, *G. intestinalis* may contribute to changes in nutrient absorption, glucose metabolism, and inflammatory signaling, while chronic metabolic inflammation may in turn shape the immunological response to the parasite (10, 21–23). These observations provide a rationale for studying giardiasis in the context of metabolic disorders and for examining whether integrated immunological and metabolic measurements may help characterize infection in this population (22, 23).

The diagnosis of giardiasis still relies primarily on conventional parasitological methods, including direct stool microscopy, which is commonly regarded as the reference approach in routine laboratory practice (24, 25). However, microscopy has several well-recognized limitations. Its diagnostic yield depends heavily on sample quality, the experience of the examiner, and, importantly, the intermittent shedding of cysts and trophozoites (25–27). As a result, single-sample testing may produce false-negative results, particularly in patients with low parasite burden or atypical clinical presentation (26–28).

Immunochromatographic and serological methods may support diagnosis, but they also have important drawbacks (28, 29). Antigen-based assays improve practicality, yet their performance may vary depending on parasite load and sampling conditions (27–29). Serological markers such as immunoglobulins can reflect exposure or host response, but individually they may not provide sufficient specificity or sensitivity for reliable discrimination of active infection in clinically complex patients (29, 30). These limitations are especially problematic in populations with metabolic disorders, where background inflammation and immune imbalance may further blur the distinction between infection-related and non-infectious alterations (14–17). With respect to these limitations, current diagnostic tools may be insufficient for precise identification of *G. intestinalis* infection in this patient group.

Although direct clinical studies addressing giardiasis specifically in adults with metabolic disorders remain limited, available evidence indicates that the clinical presentation of *G. intestinalis* infection can be heterogeneous and may overlap with gastrointestinal manifestations of metabolic conditions (31–33). In addition, giardiasis and metabolic disorders have both been associated with alterations in the gut microbiota, intestinal barrier function, and inflammatory signaling (10, 34, 35). These observations provide a rationale for investigating immunological and metabolic measurements jointly in patients with metabolic disorders, while recognizing that the direction and clinical significance of these associations remain incompletely understood (36).

The growing concept of precision diagnostics emphasizes that clinically useful disease detection may require integration of multiple biological domains rather than reliance on single markers (37–39). For giardiasis, an integrated approach may be useful for examining whether immunological and metabolic measurements jointly characterize infection, given the reported associations of giardiasis with host immune responses, intestinal function, and metabolic parameters (1, 4, 5, 22). Despite this, most diagnostic strategies remain focused on direct parasite detection or isolated laboratory parameters, without incorporating the broader host response (28, 30, 40).

Existing biological evidence suggests that *G. intestinalis* infection in adults with metabolic disorders may be associated with a combined pattern of immunological and metabolic measurements (14–17, 22). Examining these parameters jointly may therefore help characterize infection-associated patterns and generate hypotheses for subsequent investigation in larger cohorts (37–39, 41).

The primary aim of this study was to characterize immunological and metabolic measurements associated with active *G. intestinalis* infection in adults with metabolic disorders and to examine how these measurements differed according to infection status and treatment stage (22, 23, 37). Specifically, we examined differences in immunoglobulin profiles, cytokine/chemokine measurements, and metabolic parameters according to infection status and treatment stage, and evaluated which variables were associated with active infection (21, 22, 30). As an exploratory secondary analysis, we subsequently examined whether selected immunological and metabolic variables could jointly discriminate infection status using multivariable logistic regression and ROC analysis (37–39, 41).

## 2 Materials and methods

### 2.1 Study design and population

This prospective observational study was designed primarily to characterize immunological and metabolic differences associated with active *Giardia intestinalis* infection in adults with metabolic disorders. The primary analysis focused on comparisons of immunological, metabolic, and anthropometric parameters according to infection status. A follow-up assessment was performed after antiparasitic treatment in participants with *G. intestinalis* infection to reassess the selected clinical, immunological, and metabolic parameters. An exploratory multivariable analysis was subsequently performed, based on the observed between-group differences and associations, to examine whether selected immunological and metabolic variables could jointly discriminate infection status.

The planned recruitment target was 100 participants. Of these, 16 were excluded before the baseline assessment because they did not meet the predefined eligibility criteria, including malignancy (n = 5), pneumonia or another acute inflammatory condition (n = 2), infection with a parasite other than *G. intestinalis* (n = 4), and incomplete clinical or laboratory data (n = 5). Consequently, 84 participants were included in the baseline analysis, comprising 43 participants in the control group without *G. intestinalis* infection (A, Control-pre) and 41 participants with *G. intestinalis* infection (B, Giardia-pre) (**Figure 1**).

**Figure 1:**
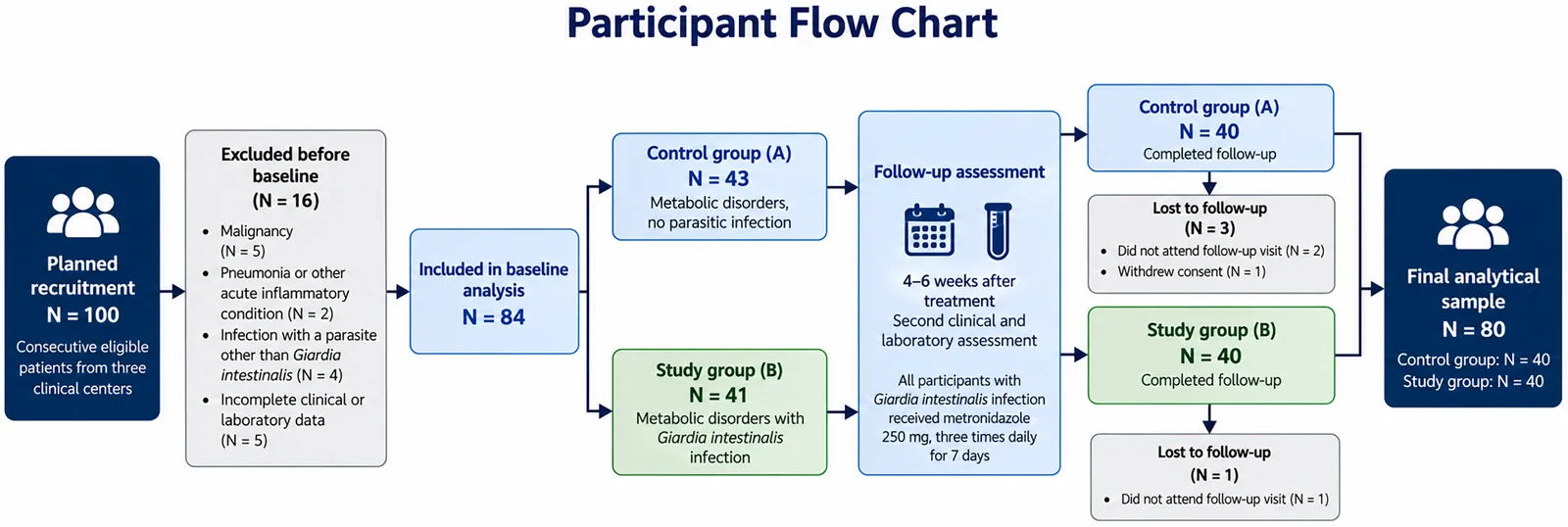
Patient recruitment flow chart. Of the 100 participants planned for recruitment, 16 were excluded before baseline assessment because they did not meet the predefined eligibility criteria, including malignancy (n=5), pneumonia or another acute inflammatory condition (n=2), infection with a parasite other than *Giardia intestinalis* (n=4), and incomplete clinical or laboratory data (n = 5). Thus, 84 participants were included in the baseline analysis, comprising 43 participants in the control group **(A)** and 41 participants in the study group **(B)** with *G. intestinalis* infection. Participants with *G. intestinalis* infection received metronidazole treatment and underwent a second clinical and laboratory assessment 4–6 weeks after treatment. During follow-up, 4 participants were not included in the final analysis: 3 from the control group and 1 from the study group. The final statistical analyses were therefore performed on 80 participants, including 40 individuals in the control group **(C)** and 40 in the study group **(D)**.

Participants were recruited by physicians at three clinical centers in Poland: AllerGen Center for Personalized Medicine in Piotrków Trybunalski; the Department of Diabetology and Internal Medicine at the Voivodeship Hospital named after Primate Cardinal Stefan Wyszyński in Sieradz; and Preveneo Health Medicine Center in Katowice. All participants had previously diagnosed metabolic disorders.

The inclusion criteria were age 40–65 years and previously diagnosed metabolic disorders, including prediabetes, type 2 diabetes mellitus diagnosed according to the American Diabetes Association (ADA) criteria, overweight, or obesity. Exclusion criteria included age <40 or >65 years, diseases coexisting with metabolic disorders that could substantially affect the study outcomes, acute inflammatory conditions, neoplastic diseases or ongoing post-treatment oncological conditions, and parasitic infections other than *G. intestinalis*. Participants were also excluded in the event of incomplete clinical or laboratory data or failure to meet the predefined eligibility criteria.

Participants were classified at baseline according to infection status into two groups: the control group without *G. intestinalis* infection (A, Control-pre) and the *G. intestinalis*-positive group (B, Giardia-pre). Baseline comparisons were performed between groups A and B. Participants with *G. intestinalis* infection received antiparasitic treatment with metronidazole (250 mg three times daily for 7 days), and a second clinical and laboratory assessment was scheduled 4–6 weeks after completion of treatment. At follow-up, repeat biological samples were collected and the relevant immunological and metabolic parameters were reassessed. Participants who completed the follow-up assessment were designated as Control-post (C) and Giardia-post (D), respectively.

Of the 43 participants in the control group at baseline, 42 returned for the follow-up assessment. One participant was lost to follow-up, and one participant had incomplete laboratory data. Of the 41 participants with *G. intestinalis* infection at baseline, all 41 returned for follow-up; however, one participant had incomplete laboratory data. Thus, 40 participants from the control group and 40 participants from the *G. intestinalis*-positive group were included in the final statistical analysis (total n = 80).

An *a priori* sample-size calculation was performed for the primary between-group comparisons. Assuming a large effect size (Cohen’s d = 0.80), a minimum of 26 participants per group was required to achieve 80% statistical power at a two-sided α = 0.05 significance level. To account for potential exclusions, incomplete data, and loss to follow-up, the recruitment target was increased to approximately 50 participants per group (100 participants in total).

The exploratory multivariable analysis was not a prespecified primary objective and was not supported by a model-specific sample-size calculation. It was undertaken subsequently, after the primary between-group analyses, to examine whether selected immunological and metabolic variables could jointly discriminate infection status. The resulting predictor combination was therefore considered hypothesis-generating and was not interpreted as a validated diagnostic or predictive model.

The study was conducted in accordance with the Declaration of Helsinki. Ethical approval was obtained from the Bioethics Committee (no. RNN/84/23/KE, 18 April 2023), and all participants provided written informed consent.

### 2.2 Diagnostic procedures

Diagnosis of *G. intestinalis* infection was established using a combined microscopic and antigen-based diagnostic approach. Three stool specimens were collected from each participant at each study stage. Each of the three specimens was examined by direct light microscopy using 0.9% sodium chloride (NaCl) solution and Lugol’s iodine solution. Each specimen was additionally tested for *G. intestinalis* antigen using a rapid immunochromatographic test (Simple Crypto-Giardia, Operon S.A., Zaragoza, Spain), according to the manufacturer’s instructions.

A participant was classified as positive for *G. intestinalis* infection when at least one of the three stool specimens showed *G. intestinalis* by direct microscopy and the antigen test was also positive. Thus, the final diagnostic classification was based on the results obtained from all three specimens rather than on a single stool sample. The same diagnostic procedure was applied at baseline and at the 4–6-week follow-up assessment after antiparasitic treatment.

### 2.3 Immunological assessment

Systemic immune response was assessed using serological, rapid immunochromatographic, and multiplex approaches. The antigen-based component of the diagnostic assessment was performed using a rapid cassette immunochromatographic test (Simple Crypto-Giardia, Operon S.A., Zaragoza, Spain), according to the manufacturer’s instructions. Levels of anti-*Giardia intestinalis* IgM and IgG antibodies were determined using a solid-phase enzyme immunoassay (Giardia lamblia IgA/IgM/IgG EIA, XEMA Co., Ltd., Ukraine), according to the manufacturer’s instructions. Circulating immune mediators were quantified using a bead-based multiplex immunoassay (Human Magnetic 30-plex Cytokine Panel, Thermo Fisher Scientific, Waltham, MA, USA; Cat. No. LHC6003M), enabling simultaneous measurement of cytokines, chemokines, and growth factors involved in Th1-, Th2-, and Th17-related pathways, as well as inflammatory and regenerative responses.

### 2.4 Metabolic and anthropometric measurements

Anthropometric assessment included body weight, body height, waist circumference, and hip circumference. Body weight was measured in kilograms (kg) using a calibrated scale, with participants wearing light clothing and no shoes. Body height was measured in centimeters (cm) using a wall-mounted stadiometer. Body mass index (BMI) was calculated as body weight (kg) divided by height squared (m²).

Waist circumference was measured in centimeters (cm) at the midpoint between the lowest palpable rib and the iliac crest, with the participant standing upright and breathing normally. Hip circumference was measured at the level of the greatest circumference of the buttocks. Waist-to-hip ratio (WHR) was calculated by dividing waist circumference by hip circumference.

Metabolic profiling was performed using venous blood samples collected after an overnight fast. The measured parameters included fasting glucose (mg/dL), fasting insulin (µIU/mL), glycated hemoglobin (HbA1c; %), total cholesterol (mg/dL), LDL cholesterol (mg/dL), HDL cholesterol (mg/dL), and triglycerides (mg/dL). Biochemical measurements were performed using Roche cobas c303 analyzers (Roche Diagnostics, Switzerland), according to the manufacturers’ instructions. Insulin resistance was estimated using the homeostasis model assessment of insulin resistance (HOMA-IR), calculated according to the standard formula: HOMA-IR = fasting insulin (µIU/mL) × fasting glucose (mg/dL)/405. Measurements were obtained at baseline and repeated at follow-up after antiparasitic treatment.

### 2.5 Statistical analysis

Statistical analyses were performed using TIBCO Statistica 13.3 software (TIBCO Software Inc., Palo Alto, CA, USA). Normality of data distribution was assessed using the Shapiro–Wilk test. Between-group differences were assessed using the Mann–Whitney U test, while paired comparisons were evaluated using the Wilcoxon signed-rank test. Correlations between immunological and metabolic parameters were analyzed using Spearman’s rank correlation coefficient. A p-value of <0.05 was considered statistically significant.

To identify variables associated with active *G. intestinalis* infection, univariable logistic regression analyses were initially performed. Variables with p < 0.05 in the univariable analyses were subsequently considered for inclusion in multivariable logistic regression models. Separate metabolic and immunological models were considered, followed by an exploratory integrated immuno-metabolic model combining variables from both domains. Variable reduction was performed using a backward stepwise procedure implemented in TIBCO Statistica 13.3. Results were expressed as odds ratios (ORs) with corresponding 95% confidence intervals (CIs).

The multivariable analysis was exploratory and was performed after the primary between-group analyses to examine whether selected immunological and metabolic variables could jointly discriminate infection status. The study was not prospectively designed for prediction-model development, and no model-specific sample-size calculation was performed before data collection. Because the predictor selection was data-driven, the resulting predictor combination was considered hypothesis-generating rather than as a definitive diagnostic or predictive model.

Diagnostic performance of individual biomarkers and multivariable models was evaluated using receiver operating characteristic (ROC) curve analysis, with calculation of the area under the curve (AUC). Optimal thresholds for individual biomarkers were determined using the Youden index.

Internal model performance was additionally assessed using non-parametric bootstrap resampling with 10,000 resamples generated by random sampling with replacement from the final analytical dataset of 80 participants, comprising 40 control participants and 40 participants with active *G. intestinalis* infection. Bootstrap distributions of model discrimination were summarized using the AUC, median AUC, and 95% bootstrap percentile confidence intervals. The bootstrap procedure was used to characterize the variability of model discrimination and was not used to calculate an optimism-corrected AUC; therefore, no separate optimism estimate or optimism-corrected AUC was derived.

As an additional internal performance assessment, the final model was evaluated using stratified 10-fold cross-validation, with preservation of the proportion of infected and control participants in each fold. The model was iteratively fitted on nine folds and evaluated on the remaining fold, with the procedure repeated until each fold had served as the validation set. The cross-validated AUC was calculated from the predictions generated for the held-out folds. Because both resampling procedures were performed within the same study cohort, their results were interpreted as measures of internal model performance and not as evidence of external diagnostic validity.

As a *post hoc* sensitivity analysis, an additional assessment based on the framework proposed by Riley et al. was performed to examine the relationship between the available sample size, model complexity, and potential overfitting (42). This analysis was conducted after the exploratory model had been identified and was not part of the original study design or a *post hoc* power calculation. It was used to assess the adequacy of the available sample in relation to the complexity of the exploratory model, rather than to justify the model or establish its validity.

## 3 Results

### 3.1 Study population characteristics

Baseline anthropometric and metabolic characteristics are presented in **Table 1**. The analyzed cohort included patients with metabolic disorders stratified according to *G. intestinalis* infection status and treatment stage.

**Table 1:** Anthropometric and metabolic characteristics of the study population.

Parameter	Control-pre (A) Mean ± SD p value	Giardia-pre (B) Mean ± SD p value	Control-post (C) Mean ± SD p value	Giardia-post (D) Mean ± SD p value
Age [years]	54.35 ± 7.85	52.83 ± 7.92 ^a^ p = 0.4129	54.35 ± 7.85 ^b^ p = 1.0000	52.83 ± 7.92 ^c^ p = 1.0000/^d^ p = 0.3581/^e^ p = 0.3574
Height [cm]	170.60 ± 9.24	171.72 ± 6.77 ^a^ p = 0.4299	170.60 ± 9.24 ^b^ p = 1.0000	171.72 ± 6.77 ^c^ p = 1.0000/^d^ p = 0.4416/^e^ p = 0.4406
Weight [kg]	83.11 ± 14.90	81.56 ± 13.47 ^a^ p = 0.2657	85.28 ± 14.12 ^b^ p = 0.4943	82.14 ± 13.40 ^c^ p = 0.6392/^d^ p = 0.0986/^e^ p = 0.3599
Waist circumference [cm]	99.55 ± 10.66	96.10 ± 10.75 ^a^ p = 0.0060	99.55 ± 10.66 ^b^ p = 1.0000	94.28 ± 10.28 ^c^ p = 0.4069/^d^ p = 0.0102/^e^ p = 0.0099
Hip circumference [cm]	106.47 ± 9.83	105.00 ± 9.50 ^a^ p = 0.4444	106.30 ± 9.94 ^b^ p = 0.8242	105.10 ± 9.83 ^c^ p = 0.9460/^d^ p = 0.5605/^e^ p = 0.4595
WHR	0.94 ± 0.04	0.92 ± 0.07 ^a^ p = 0.0385	0.94 ± 0.04 ^b^ p = 0.8586	0.92 ± 0.14 ^c^ p = 0.6465/^d^ p = 0.0262/^e^ p = 0.0289
BMI	28.48 ± 4.02	23.71 ± 3.56 ^a^ p < 0.0001	29.40 ± 5.16 ^b^ p = 0.6236	23.88 ± 3.55 ^c^ p = 0.6824/^d^ p < 0.0001/^e^ p < 0.0001
Glucose [mg/dl]	154.69 ± 89.11	97.58 ± 24.25 ^a^ p = 0.0002	156.46 ± 87.94 ^b^ p = 0.7987	111.71 ± 26.68 ^c^ p = 0.0002/^d^ p = 0.0081/^e^ p = 0.0231
HbA1c [%]	8.69 ± 2.88	6.23 ± 1.67 ^a^ p < 0.0001	8.86 ± 2.87 ^b^ p = 0.6166	6.62 ± 1.61 ^c^ p = 0.0395/^d^ p = 0.0002/^e^ p = 0.0008
Total cholesterol [mg/dl]	204.23 ± 54.19	213.36 ± 52.83 ^a^ p = 0.8177	205.35 ± 53.26 ^b^ p = 0.8814	213.57 ± 52.02 ^c^ p = 0.9348/^d^ p = 0.7146/^e^ p = 0.6896
HDL [mg/dl]	51.56 ± 16.26	58.39 ± 16.33 ^a^ p = 0.0565	51.94 ± 14.91 ^b^ p = 0.1305	58.23 ± 16.33 ^c^ p = 0.9985/^d^ p = 0.0661/^e^ p = 0.0519
LDL [mg/dl]	124.51 ± 40.70	127.00 ± 55.52 ^a^ p = 0.9804	124.51 ± 40.70 ^b^ p = 1.0000	127.00 ± 55.52 ^c^ p = 1.0000/^d^ p = 0.9808/^e^ p = 1.0000
Triglycerides [mg/dl]	152.70 ± 75.61	128.30 ± 65.71 ^a^ p = 0.1776	153.28 ± 74.17 ^b^ p = 0.0815	133.13 ± 65.76 ^c^ p = 0.5800/^d^ p = 0.2057/^e^ p = 0.2272
TG/HDL	3.75 ± 3.79	2.57 ± 1.90 ^a^ p = 0.0818	3.50 ± 2.93 ^b^ p = 0.3613	2.65 ± 1.90 ^c^ p = 0.7039/^d^ p = 0.1225/^e^ p = 0.1296
Insulin [µIU/ml]	21.08 ± 22.63	14.36 ± 11.18 ^a^ p = 0.2513	19.87 ± 22.32 ^b^ p = 0.7545	13.28 ± 10.23 ^c^ p = 0.6372/^d^ p = 0.2684/^e^ p = 0.1296
HOMA-IR	10.15 ± 23.70	3.45 ± 2.61 ^a^ p = 0.0291	9.99 ± 23.73 ^b^ p = 0.8738	3.66 ± 2.80 ^c^ p = 0.2014/^d^ p = 0.0561/^e^ p = 0.0347
CRP [mg/L]	4.98 ± 8.52	3.82 ± 9.45 ^a^ p = 0.0712	3.19 ± 7.56 ^b^ p = 0.0185	2.31 ± 3.74 ^c^ p = 0.0675/^d^ p = 0.5510/^e^ p = 0.0011

Data are presented as mean ± SD. Superscript letters indicate the following pairwise comparisons: ^a^ A vs. B; ^b^ A vs. C; ^c^ B vs. D; ^d^ C vs. D; ^e^ A vs. D. A p-value of <0.05 was considered statistically significant.

BMI, body mass index; WHR, waist-to-hip ratio; HOMA-IR, homeostatic model assessment of insulin resistance; HbA1c, glycated hemoglobin; LDL, low-density lipoprotein; HDL, high-density lipoprotein. Note: Data are presented as mean ± SD. ^a^ p – vs. Control-pre (A); ^b^ p – vs. Control-pre (A); ^c^ p – vs. Control-post (C); ^d^ p – vs. Giardia-pre (B); ^e^ p – vs. Control-pre (A).

There were no significant differences in age, height, or body weight between groups. However, significant differences were observed in selected anthropometric and metabolic parameters. At baseline, patients with active *G. intestinalis* infection (group B) exhibited lower fasting glucose levels, BMI, and HOMA-IR values compared with non-infected controls (group A) (p=0.0002, p<0.0001, and p=0.0291, respectively). Additionally, waist circumference and WHR were significantly lower in the infected group (p=0.0060 and p=0.0385, respectively). Following antiparasitic treatment (group D), these parameters increased and approached levels observed in control subjects (group C). Significant differences between pre- and post-treatment measurements were observed for glucose, HbA1c, and HOMA-IR, indicating changes in these metabolic parameters between the pre-treatment and post-treatment assessments.

No statistically significant differences were found in lipid profile parameters (total cholesterol, LDL, HDL, triglycerides) between groups, although a trend toward lower triglyceride levels during active infection was observed. Detailed comparisons are shown in **Table 1**.

### 3.2 Immunological differences between groups

Differences in humoral immune response between study groups are summarized in **Table 2**.

**Table 2:** Frequency of anti-*Giardia intestinalis* IgM and IgG antibodies across study groups.

Group	IgM+ N (%)	IgM− N (%)	IgG+ N (%)	IgG− N (%)
Control-pre (A)	0 (0)	40 (100)	11 (27.5)	29 (72.5)
Giardia-pre (B)	26 (65)	14 (35)	29 (72.5)	11 (27.5)
Control-post (C)	0 (0)	40 (100)	8 (20)	32 (80)
Giardia-post (D)	9 (22.5)	31 (77.5)	33 (82.5)	7 (17.5)

Group A, control participants before treatment; group B, participants with active *G. intestinalis* infection before treatment; group C, control participants at follow-up; group D, participants with *G. intestinalis* infection after antiparasitic treatment. Data are presented as n (%). A p-value of <0.05 was considered statistically significant.

At baseline, IgM antibodies were detected exclusively in patients with active *G. intestinalis* infection (group B), with a positivity rate of 65%, whereas no IgM-positive cases were observed in the control group (group A). In contrast, IgG antibodies were present in both groups, although significantly more frequently in infected individuals (72.5% vs 27.5%, p<0.00001). After antiparasitic treatment, a marked shift in immunological profile was observed. In the Giardia-post group (D), the frequency of IgM positivity decreased to 22.5%, whereas IgG seropositivity remained high (82.5%). In the control follow-up group (C), IgM antibodies remained undetectable, while IgG positivity was observed in 20% of participants.

Differences between groups remained statistically significant after treatment (p=0.00465 for IgM; p<0.0001 for IgG).

### 3.3 Metabolic alterations associated with infection

Active *Giardia intestinalis* infection was associated with significant alterations in metabolic parameters. Compared with non-infected controls (group A), infected patients at baseline (group B) exhibited significantly lower BMI, fasting glucose levels, and HOMA-IR values (p<0.0001, p=0.0002, and p=0.0291, respectively).

No significant differences were observed in lipid profile parameters, although a decreasing trend in triglyceride levels was noted.

Following antiparasitic treatment (group D), BMI, glucose, and HOMA-IR values increased, approaching levels observed in control subjects (group C).

### 3.4 Cytokine and chemokine profiling

Profiles of cytokines, chemokines, and growth factors across study groups are presented in **Figure 2**. At baseline, patients with active infection (group B) exhibited significantly lower concentrations of MCP-1, RANTES, IL-8, and HGF compared with controls (group A) (p=0.0168, p=0.0031, p<0.0001, and p=0.0005, respectively).

**Figure 2:**
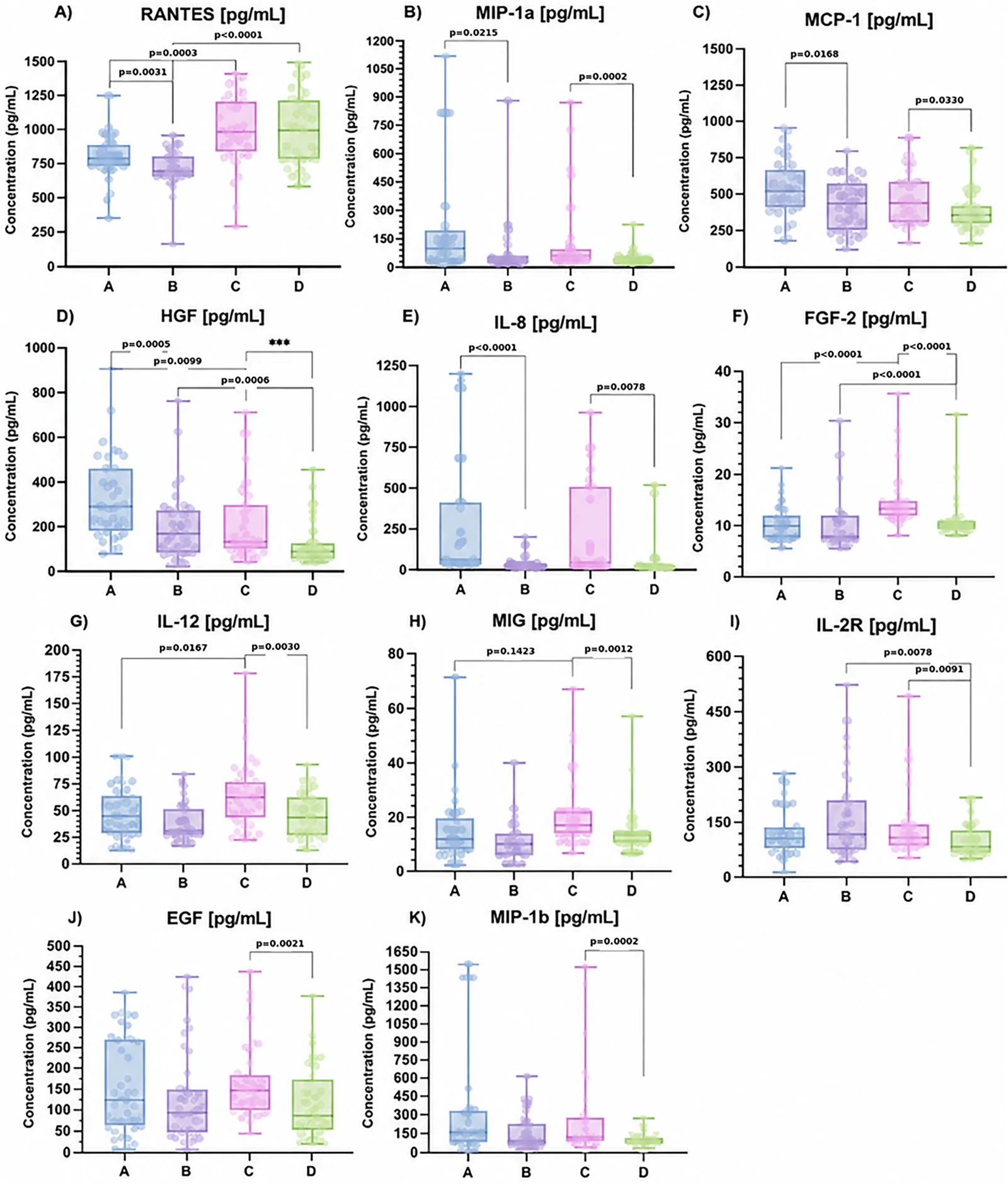
Comparison of concentrations of selected cytokines, chemokines, and growth factors in patients with metabolic disorders with and without *Giardia intestinalis* infection, before and after antiparasitic treatment. Box plots **(A–K)** present the distribution of selected immunological mediators (pg/mL) across study groups: **(A)** – control before treatment (patients with metabolic disorders without infection), **(B)** – infected before treatment (*G. intestinalis*), **(C)** – control at follow-up, and **(D)** – infected after antiparasitic treatment.

After treatment (group D), a significant increase in these mediators was observed, particularly for HGF (p=0.0006), MCP-1 (p=0.0330), and IL-8, while selected markers remained significantly different from controls.

Comparisons between control groups (A vs C) also revealed increased levels of selected cytokines during follow-up, including IL-12, RANTES, and HGF.

### 3.5 Correlation between metabolic and immunological parameters

Correlation analysis revealed significant associations between metabolic and immunological parameters (**Table 3**). In patients with active infection (group B), MCP-1 positively correlated with glucose levels and BMI, and was additionally associated with inflammatory markers such as CRP, indicating associations between selected chemokine, inflammatory, and metabolic measurements. After treatment (group D), the number and strength of correlations decreased, although associations involving IL-12, IL-2R, and insulin resistance markers (HOMA-IR and fasting insulin) persisted.

**Table 3:** Spearman rank correlations between immunological and metabolic parameters according to *G. intestinalis* infection status and treatment stage.

Comparison	Parameter	Cytokine	rho A	rho B	rho C	rho D	p
Control-pre vs Giardia-pre (A vs B)	BMI	RANTES	-0.34	0.12			0.0321
	BMI	HGF	-0.32	0.27			0.0441
	Glucose [mg/dl]	IL-12	-0.14	0.34			0.0323
	LDL [mg/dl]	Eotaxin	-0.32	0.29			0.0493
	LDL [mg/dl]	MCP-1	-0.24	0.38			0.0158
	Triglycerides [mg/dl]	IL-12	0.35	0.08			0.0270
	TG/HDL	EGF	0.25	-0.33			0.0394
	WHR	RANTES	-0.33	-0.08			0.0352
	WHR	IL-8	-0.03	0.41			0.0093
	Weight [kg]	RANTES	-0.42	0.13			0.0071
	Weight [kg]	HGF	-0.46	0.13			0.0029
	CRP	MIP-1b	0.35	0.33			0.0283
	CRP	HGF	0.24	0.48			0.0016
	Hip circumference [cm]	MCP-1	-0.42	-0.01			0.0065
	Hip circumference [cm]	EGF	-0.23	-0.33			0.0350
	Waist circumference [cm]	RANTES	-0.47	0.06			0.0024
Control-post vs Giardia-post (C vs D)	BMI	MIP-1a			0.35	0.17	0.0285
	BMI	MCP-1			0.32	-0.3	0.0443
	BMI	HGF			0.36	-0.23	0.0212
	Triglycerides [mg/dl]	MIP-1b			0.14	-0.36	0.0236
	Triglycerides [mg/dl]	MCP-1			0.04	0.31	0.0495
	TG/HDL	MIG			0.12	-0.32	0.0473
	TG/HDL	MIP-1b			0.06	-0.41	0.0081
	TG/HDL	MCP-1			0.03	0.33	0.0348
	WHR	MIP-1b			-0.33	0.03	0.0403
	WHR	IL-8			-0.37	-0.11	0.0191
	Weight [kg]	FGF-2			-0.36	0.11	0.0243
	Weight [kg]	Eotaxin			0.34	-0.16	0.0314
	Weight [kg]	MCP-1			0.45	-0.31	0.0040
	CRP	IL-12			0.06	-0.34	0.0298
Control-pre vs Control-post (A vs C)	BMI	RANTES	-0.34		-0.06		0.0321
	BMI	MIP-1a	-0.21		0.35		0.0285
	BMI	MCP-1	-0.15		0.32		0.0443
	BMI	HGF	-0.32		0.36		0.0212
	LDL [mg/dl]	Eotaxin	-0.32		0.12		0.0493
	Triglycerides [mg/dl]	IL-12	0.35		-0.0		0.0270
	WHR	RANTES	-0.33		-0.22		0.0352
	WHR	MIP-1b	0.03		-0.33		0.0403
	WHR	IL-8	-0.03		-0.37		0.0191
	Weight [kg]	FGF-2	-0.2		-0.36		0.0243
	Weight [kg]	RANTES	-0.42		-0.26		0.0071
	Weight [kg]	Eotaxin	0.04		0.34		0.0314
	Weight [kg]	MCP-1	-0.14		0.45		0.0040
	Weight [kg]	HGF	-0.46		0.22		0.0029
	CRP	RANTES	-0.04		-0.35		0.0247
	CRP	MIP-1b	0.35		0.14		0.0283
	CRP	HGF	0.24		0.56		0.0002
	CRP	IL-2R	0.04		0.31		0.0481
	HOMA-IR	IL-12	-0.14		-0.46		0.0030
	HOMA-IR	IL-2R	0.11		-0.36		0.0237
	Insulin [µU/ml]	IL-12	-0.09		-0.5		0.0012
	Hip circumference [cm]	Eotaxin	0.0		0.4		0.0112
	Hip circumference [cm]	MCP-1	-0.42		0.29		0.0065
	Waist circumference [cm]	RANTES	-0.47		-0.12		0.0024
	Waist circumference [cm]	EGF	-0.32		0.13		0.0419
Giardia-pre vs Giardia-post (B vs D)	Glucose [mg/dl]	IL-12		0.34		0.24	0.0323
	LDL [mg/dl]	MCP-1		0.38		0.07	0.0158
	Triglycerides [mg/dl]	MIP-1b		0.22		-0.36	0.0236
	Triglycerides [mg/dl]	MCP-1		0.06		0.31	0.0495
	Triglycerides [mg/dl]	MIG		0.07		-0.32	0.0473
	TG/HDL	MIP-1b		0.14		-0.41	0.0081
	TG/HDL	MCP-1		0.02		0.33	0.0348
	TG/HDL	EGF		-0.33		-0.19	0.0394
	WHR	IL-8		0.41		-0.11	0.0093
	CRP	IL-12		0.07		-0.34	0.0298
	CRP	MIP-1b		0.33		-0.13	0.0367
	CRP	HGF		0.48		0.15	0.0016
	CRP	IL-2R		0.18		-0.44	0.0044
	Hip circumference [cm]	EGF		-0.33		0.19	0.0350

Data are presented as Spearman’s correlation coefficient (ρ) and corresponding p-values. Group A, control participants before treatment; group B, participants with active *G. intestinalis* infection before treatment; group C, control participants at follow-up; group D, participants with *G. intestinalis* infection after antiparasitic treatment. A p-value of <0.05 was considered statistically significant.

Overall, correlation analysis identified associations between selected immunological and metabolic measurements that differed according to infection status and treatment stage.

### 3.6 Exploratory regression analysis of variables associated with infection

To identify variables associated with active *Giardia intestinalis* infection, logistic regression analysis was performed (**Table 4**). In univariate models, several immunological parameters were significantly associated with infection status, including IgM antibodies, IL-12, IL-8, RANTES, IL-2R, HGF, MCP-1, and MIP-1β. Among these, IgM antibodies showed the strongest association with infection status in the univariable analysis. Detailed regression results are presented in **Table 5**.

**Table 4:** Univariable and multivariable logistic regression analyses of immunological and metabolic parameters associated with active *G. intestinalis* infection.

Parameter	Univariate logistic regression			Multivariate logistic regression		
	OR	p (Wald)	95% CI	OR	p (Wald)	95% CI
FGF-2	0.945	0.0756	(0.888, 1.006)	–	–	–
IL-12	0.975	0.0006	(0.961, 0.989)	0.977	0.0165	(0.959, 0.996)
RANTES	0.999	0.2268	(0.998, 1.001)	0.992	0.0002	(0.988, 0.996)
MIP-1a	0.997	0.0100	(0.995, 0.999)	–	–	–
MIP-1b	0.997	0.0039	(0.995, 0.999)	–	–	–
MCP-1	0.997	0.0007	(0.995, 0.999)	0.997	0.0405	(0.995, 1.0)
EGF	0.996	0.0158	(0.993, 0.999)	–	–	–
HGF	0.996	0.0002	(0.993, 0.998)	0.996	0.0223	(0.992, 0.999)
IL-2R	1.000	0.9833	(0.996, 1.004)	1.013	0.0015	(1.005, 1.021)
MIG	0.955	0.0106	(0.922, 0.989)	–	–	–
IL-8	0.995	0.0001	(0.993, 0.998)	0.995	0.0021	(0.992, 0.998)

OR, odds ratio; CI, confidence interval. Univariable logistic regression analyses were initially performed for individual parameters. Variables with p<0.05 in the univariable analyses were simultaneously entered into the initial multivariable logistic regression model. Variable selection was performed using an automated backward stepwise procedure, with variables removed according to the predefined statistical significance criterion. ORs are presented with 95% CIs. A p-value of <0.05 was considered statistically significant.

**Table 5:** Receiver operating characteristic (ROC) curve analysis of individual immunological (A) and metabolic (B) parameters for discrimination of active *G. intestinalis* infection.

Parameter	AUC	95% CI	p value	Sensitivity (%)	Specificity (%)	Youden index	Cut-off
(a)							
IL-2R	0.569	(0.455, 0.684)	0.1940	45.0	76.7	0.217	148.93
HGF	0.487	(0.390, 0.584)	0.8070	55.0	51.7	0.067	155.78
MCP-1	0.441	(0.339, 0.552)	0.2690	45.0	62.5	0.075	486.48
EGF	0.422	(0.314, 0.527)	0.1400	7.5	99.2	0.067	394.34
MIP-1b	0.407	(0.299, 0.516)	0.0801	27.5	75.8	0.033	279.258
IL-8	0.354	(0.260, 0.448)	0.0057	0.0	100.0	<0.001	1197.97
MIP-1a	0.351	(0.246, 0.459)	0.0048	5.0	99.2	0.042	881.89
IL-12	0.347	(0.259, 0.444)	0.0039	100.0	7.5	0.075	15.90
FGF-2	0.322	(0.213, 0.439)	0.0010	13.2	93.3	0.064	20.28
MIG	0.311	(0.216, 0.415)	0.0003	5.0	95.8	0.008	39.80
RANTES	0.222	(0.150, 0.297)	<0.0001	97.5	3.3	0.008	513.39
IgM	0.788	(0.705, 0.863)	<0.0001	65.0	92.5	0.575	1.00
IgG	0.646	(0.557, 0.729)	0.0015	72.5	56.7	0.292	1.00
(b)							
HDL [mg/dl]	0.587	(0.484, 0.690)	0.0999	67.5	53.3	0.208	55.00
CRP	0.534	(0.436, 0.633)	0.5156	67.5	49.2	0.167	1.05
Total cholesterol [mg/dl]	0.515	(0.413, 0.614)	0.7751	82.5	30.8	0.133	176.10
LDL [mg/dl]	0.499	(0.391, 0.608)	0.9824	12.5	95.8	0.083	211.00
Insulin [µU/ml]	0.468	(0.369, 0.568)	0.5452	100.0	2.5	0.025	1.70
Triglycerides [mg/dl]	0.421	(0.320, 0.523)	0.1338	5.0	96.7	0.017	321.30
WHR	0.410	(0.306, 0.519)	0.0897	10.0	94.2	0.042	1.02
TG/HDL	0.410	(0.306, 0.516)	0.0876	0.0	100.0	<0.001	21.98
HOMA-IR	0.399	(0.300, 0.500)	0.0560	0.0	100.0	<0.001	148.80
BMI	0.272	(0.189, 0.364)	<0.0001	100.0	1.7	0.017	18.29
HbA1c [%]	0.246	(0.163, 0.341)	<0.0001	0.0	100.0	<0.001	15.60
Glucose [mg/dl]	0.241	(0.167, 0.324)	<0.0001	0.0	100.0	<0.001	495.00

AUC, area under the ROC curve; CI, confidence interval. Sensitivity and specificity are expressed as percentages. The optimal cut-off value for each parameter was determined using the Youden index. AUC values are presented with 95% CIs. A p-value of <0.05 was considered statistically significant.

In multivariable analysis, IgM, IL-12, IL-8, RANTES, and IL-2R remained associated with infection status. These findings indicate that both humoral and cytokine-related measurements were associated with infection status. The results of the multivariable analysis were used to inform the subsequent exploratory evaluation of joint discriminatory performance.

### 3.7 Discriminatory performance of individual markers

The discriminatory performance of individual immunological and metabolic markers was evaluated using receiver operating characteristic (ROC) curve analysis (**Table 5**, **Figure 3**). Among all tested parameters, IgM antibodies demonstrated the highest AUC (approximately 0.788), indicating greater discriminatory performance than the other individual markers evaluated.

**Figure 3:**
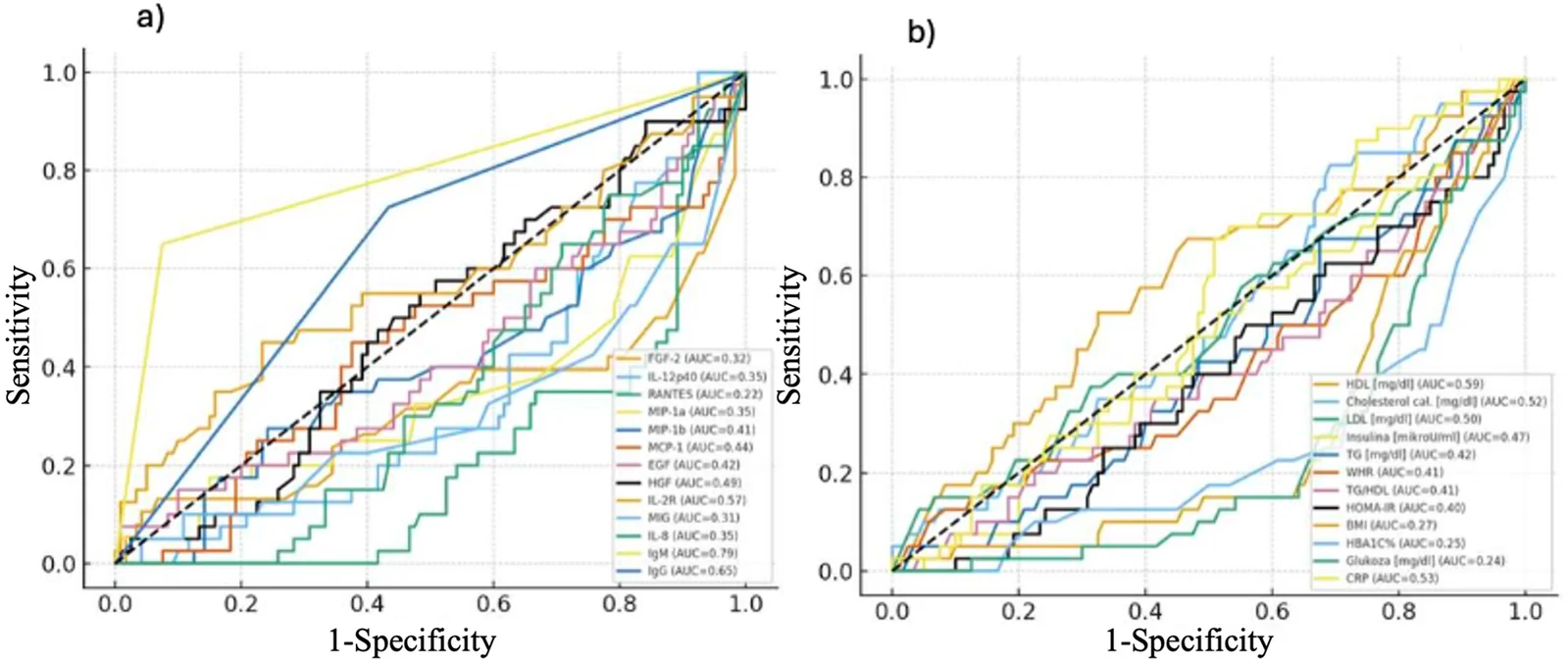
Receiver operating characteristic **(ROC)** curve analysis of immunological **(A)** and metabolic **(B)** markers for the identification of active *Giardia intestinalis* infection in patients with metabolic disorders.

Other immunological markers, including IgG and IL-2R, showed moderate discrimination, whereas most cytokines and chemokines had limited discriminatory performance. Metabolic parameters also showed limited discrimination, with AUC values generally below 0.600. These findings provided a rationale for exploratory evaluation of whether combinations of immunological and metabolic variables could provide additional discriminatory information beyond individual measurements.

### 3.8 Multivariable logistic regression models

Based on the associations observed in the preceding analyses, an exploratory multivariable logistic regression analysis was performed to examine whether selected immunological and metabolic variables could jointly discriminate participants with active *G. intestinalis* infection from controls.

The analysis resulted in an integrated set of six variables: glucose, BMI, IgM, RANTES, IL-12, and IL-2R. The apparent AUC of the exploratory integrated model was 0.953. The corresponding odds ratios (ORs), 95% confidence intervals (CIs), and p-values are presented in **Table 6**.

**Table 6:** Domain-specific and combined logistic regression models for the prediction of active *G. intestinalis* infection in patients with metabolic disorders aged 40–65 years.

Parameter	Metabolic model		Immunological model		Combined model	
	OR (95% CI)	P (Wald)	OR (95% CI)	P (Wald)	OR (95% CI)	P (Wald)
Glucose [mg/dL]	0.970 (0.951–0.989)	0.0020	–	–	0.968 (0.942–0.995)	0.0207
BMI	0.854 (0.769–0.947)	0.0029	–	–	0.795 (0.668–0.946)	0.0096
IgM	–	–	42.058 (11.509–153.690)	<0.0001	24.034 (5.602–103.122)	<0.0001
RANTES	–	–	0.995 (0.993–0.998)	0.0008	0.994 (0.991–0.998)	0.0011
IL-12	–	–	0.955 (0.926–0.984)	0.0030	0.956 (0.924–0.988)	0.0075
IL-2R	–	–	1.011 (1.003–1.018)	0.0035	1.016 (1.007–1.025)	0.0004

The metabolic and immunological models were developed separately using their respective candidate variables and backward stepwise selection. The combined immuno-metabolic model was constructed by considering the metabolic and immunological candidate variables together and applying the predefined backward stepwise procedure. Model performance is summarized by Akaike information criterion (AIC) and area under the receiver operating characteristic curve (AUC).

AIC, Akaike information criterion; AUC, area under the receiver operating characteristic curve; BMI, body mass index; IgM, immunoglobulin M; IL-2R, interleukin-2 receptor; RANTES, regulated upon activation, normal T-cell expressed and secreted.

The apparent AUCs of the metabolic and immunological models were 0.808 and 0.926, respectively. These differences in apparent AUC are presented descriptively, as no formal statistical comparison between the AUCs was performed. Model performance was therefore further examined using internal resampling procedures.

### 3.9 Internal performance assessment and predictor-selection stability

Integrated model performance was further assessed using internal resampling procedures. The model showed an apparent AUC of 0.953. In a 10,000-resample bootstrap analysis, the mean AUC was 0.953 and the median AUC was 0.955, with a 95% bootstrap percentile interval of approximately 0.908–0.984. The bootstrap results were used to characterize the variability of discrimination of the specified final model and were not interpreted as an optimism-corrected AUC.

In addition, the model was evaluated using stratified 10-fold cross-validation, with preservation of the proportion of infected and control participants in each fold. The reported cross-validated AUC was 0.954. Because both procedures were performed within the same study cohort, these estimates should not be interpreted as evidence of external diagnostic validity.

The bootstrap assessment of AUC does not establish stability of the individual predictor-selection process. To further explore this issue, a separate retrospective sensitivity analysis examined predictor-selection stability across 10,000 bootstrap samples using the same candidate-predictor framework. Selection frequencies differed substantially between predictors: IgM was retained in 97.1% of resamples, BMI in 88.9%, IL-2R in 60.3%, IL-12 in 30.9%, RANTES in 29.3%, and glucose in 22.1%. Thus, the individual components of the final model showed different degrees of selection stability across resamples.

Overall, the specified model showed consistently high discrimination across the internal resampling procedures, but these analyses did not establish the stability or generalizability of the individual predictor set. The predictor-selection analysis was a retrospective sensitivity analysis and was not part of the original model-development procedure.

### 3.10 Retrospective assessment of sample-size adequacy

The final integrated model included six predictor parameters and was developed in 80 participants, including 40 participants with active infection. Thus, the final model was based on 40 outcome events for six predictor parameters (6.7 events per parameter).

The retrospective sensitivity assessment based on the Riley framework demonstrated that the required sample size was substantially larger than the available cohort across all examined scenarios (43).

These estimates indicate that the present dataset was relatively small for development of a multivariable prediction model and support the exploratory interpretation of the integrated model.

## 4 Discussion

### 4.1 Key findings

The present study identified differences in immunological and metabolic measurements according to active *Giardia intestinalis* infection status in adults with metabolic disorders. Patients with active giardiasis exhibited significantly lower concentrations of IL-12 and IL-8 together with reduced levels of MCP-1, RANTES, and HGF. These differences are compatible with alterations in systemic immune responses during active infection, although the cross-sectional nature of the analysis does not allow causal mechanisms to be established. These immunological differences were accompanied by lower BMI, fasting glucose, HbA1c, and HOMA-IR values. Thus, active *G. intestinalis* infection was associated with a distinct metabolic profile in the study population; however, the observed associations do not establish that infection directly caused these metabolic differences. Similar immunological and epithelial alterations have previously been described in experimental and clinical studies of giardiasis (4, 22, 43).

Importantly, the post-treatment assessment showed partial normalization of several metabolic and immunological parameters. Following antiparasitic treatment, concentrations of several cytokines and chemokines increased toward values observed in the control group, whereas metabolic indices such as glucose and HbA1c also shifted toward baseline levels. These findings indicate that the immunological and metabolic measurements differed between the pre-treatment and post-treatment assessments. However, because the study was observational and other factors may have contributed to changes over time, these findings should not be interpreted as demonstrating a direct causal effect of parasite eradication.

An additional exploratory finding was that a combination of immunological and metabolic variables showed high apparent discriminatory performance within the present cohort. The integrated model combining IgM, IL-2R, IL-12, RANTES, BMI, and glucose showed an apparent AUC of 0.953, compared with AUCs of 0.808 for the metabolic model and 0.926 for the immunological model. These differences are presented descriptively and should not be interpreted as evidence of statistically significant superiority, as no formal statistical comparison between the AUCs was performed. Because the predictor selection was data-driven and the model was not developed as a prespecified prediction model, these findings should be considered exploratory.

### 4.2 Immunological mechanisms

The immunological profile observed in the present study is compatible with previously described alterations in host immune responses during giardiasis. However, the present data demonstrate associations between infection status and circulating immune markers and do not establish that *G. intestinalis* actively modulates these responses or that such changes facilitate parasite persistence. Previous experimental and clinical studies have shown that protective immunity against *Giardia* is largely dependent on Th1-associated responses involving IL-12, IFN-γ, macrophage activation, and recruitment of inflammatory effector cells (9, 44, 45). In the current study, reduced concentrations of IL-12 and IL-8 were observed in actively infected individuals. These findings are compatible with differences in Th1-related and innate inflammatory responses, although the underlying mechanisms cannot be determined from the present data.

IL-12 plays a central role in differentiation of naive T lymphocytes toward the Th1 phenotype and stimulates IFN-γ production, thereby enhancing cellular antiparasitic immunity. The markedly decreased IL-12 concentrations observed in infected patients are consistent with previous reports describing alterations in Th1-related responses during giardiasis (9, 45). However, the present findings do not demonstrate whether these changes result from parasite-mediated immune modulation or from other infection-related or host-related factors. Similarly, IL-8 concentrations were reduced during active infection. Given the role of IL-8 in neutrophil recruitment, this finding is compatible with differences in inflammatory signaling, but circulating IL-8 concentrations alone cannot establish suppression of local intestinal innate immune responses.

At the same time, alterations in chemokines such as MCP-1 and RANTES are compatible with differences in chemokine-mediated immune signaling. MCP-1 is involved in monocyte recruitment and tissue inflammatory signaling, whereas RANTES participates in lymphocyte trafficking and chronic inflammatory regulation. Reduced systemic levels of these mediators during active infection, followed by changes after treatment, are compatible with a dynamic pattern of immune-marker variation across infection and follow-up. However, these observations do not establish that *G. intestinalis* induces transient immunomodulation or determine the mechanisms underlying these changes. Similar mechanisms of immune evasion and epithelial immune modulation have been proposed previously (4, 22, 46).

The observed increase in IL-2R after treatment may be indicative of changes in T-cell-related immune activity during follow-up. However, this temporal association does not establish that parasite eradication was responsible for the observed change. Together, these findings are consistent with differences in immune balance during active *G. intestinalis* infection, but the present data do not establish whether these changes contribute to impaired parasite clearance, parasite persistence, or modulation of the host immune response (22, 43).

In addition, serological findings remained significantly different between infected and non-infected individuals after treatment (IgM: p=0.00465; IgG: p<0.0001). This observation is consistent with the persistence of differences in antibody profiles after treatment. However, the present study cannot determine whether the observed IgM and IgG patterns reflect ongoing infection, recent exposure, or persistence of antibody responses after infection (9, 45).

### 4.3 Clinical implications

The potential clinical relevance of the present findings warrants further investigation, particularly in populations with underlying metabolic disorders. Conventional diagnostic methods for giardiasis, including microscopy and rapid antigen detection tests, are characterized by limited sensitivity and may generate false-negative results due to intermittent cyst shedding, low parasite burden, and operator-dependent interpretation (4, 43). This issue may be especially relevant in patients with metabolic syndrome, obesity, or insulin resistance, in whom gastrointestinal symptoms are often nonspecific and may overlap with manifestations of metabolic disease. The present findings suggest that immunological and metabolic measurements may provide complementary information for characterizing infection, but they do not establish that these measurements improve diagnosis beyond conventional parasitological methods.

The integrated immuno-metabolic model showed an apparent AUC of 0.953 in the present cohort. This result supports further investigation of the identified variable combination rather than immediate clinical application. Whether these variables provide additional information beyond conventional diagnostic approaches, or whether they could have value for diagnosis or treatment monitoring, remains to be determined in prospective studies with independent validation.

The exploratory nature of the present findings also precludes conclusions regarding improved detection, reduced false-negative diagnoses, earlier diagnosis, or clinical treatment decisions. Such questions require dedicated prospective diagnostic-accuracy and clinical-utility studies.

### 4.4 Comparison with previous studies

The present findings are broadly consistent with previous experimental and clinical observations showing that *Giardia* infection is associated with changes in host immune responses. Experimental studies have demonstrated that *Giardia* can modify cytokine and chemokine signaling at the intestinal epithelial and immune-cell interface. Roxström-Lindquist et al. reported a distinct chemokine response in *Giardia*-exposed human Caco-2 cells, characterized by increased expression of CCL2 (MCP-1), CCL20, CXCL1, CXCL2, and CXCL3 (46). Subsequent experimental studies demonstrated that *Giardia* can interfere with chemokine-mediated recruitment of inflammatory cells. In particular, Cotton et al. showed that *Giardia duodenalis* cathepsin B proteases degrade epithelial IL-8 and attenuate IL-8-dependent neutrophil chemotaxis (45). These observations provide a mechanistic context for the altered chemokine and IL-8 concentrations observed in the present study.

The findings concerning IL-12 are also in agreement with previous reports. Studies of human giardiasis have documented alterations in IL-12 concentrations, including lower serum IL-12 levels in patients with *G. intestinalis* infection (46). Experimental studies have additionally demonstrated that *Giardia* antigens can modulate IL-12 production by dendritic cells, indicating that this cytokine is involved in the host response to the parasite (44). Thus, the lower IL-12 concentrations observed in the present cohort are consistent with previously described alterations in Th1-related immune responses during giardiasis.

Importantly, however, the chemokine findings of the present study provide additional clinical data to the existing literature. Previous human studies have investigated MCP-1 and other cytokines in relation to *Giardia* infection, but these studies have predominantly focused on fecal immune markers in young children. For example, Long et al. measured fecal MCP-1, IL-8, IL-4, IL-5, IL-6, IL-10, TNF-α, and IFN-γ in Mexican children and found that MCP-1 concentrations were associated with the duration of *G. intestinalis* infection (47). In contrast, the present study evaluated circulating MCP-1 and RANTES together with other immunological mediators in adults with metabolic disorders and active *G. intestinalis* infection. These differences in population, specimen type, and biomarker profile limit direct comparison between studies but indicate that systemic immune responses in adults with metabolic disorders warrant further investigation.

A further finding of interest is the reduction in circulating HGF concentrations during active *G. intestinalis* infection and their increase following antiparasitic treatment. Although HGF has been investigated as a marker of intestinal injury and infectious gastroenteritis, including in human patients, these studies have not specifically characterized circulating HGF in adults with active giardiasis (48). The present findings therefore suggest that HGF may be associated with the systemic response observed during active infection, although this interpretation requires confirmation in independent cohorts.

The metabolic findings further distinguish the present study from much of the previous literature. Most earlier studies of giardiasis have focused on parasitological, clinical, mucosal, or immunological outcomes, whereas our study specifically examined immunological alterations in the context of pre-existing metabolic disorders and simultaneously evaluated metabolic parameters. The observed associations between MCP-1, RANTES, and metabolic variables provide an additional dimension that has not been well characterized in previous clinical studies of giardiasis. The combination of immunological and metabolic measurements therefore represents a potentially useful framework for further investigation in this population.

Finally, the present study explored whether serological, immunological, anthropometric, and metabolic measurements could be considered jointly in relation to infection status. The integrated model showed an apparent AUC of 0.953, compared with 0.808 for the metabolic model and 0.926 for the immunological model. These differences are descriptive, as no formal statistical comparison between the AUCs was performed. The multivariable combination was identified as part of the exploratory secondary analysis and should therefore be regarded as hypothesis-generating rather than as a validated diagnostic framework.

### 4.5 Strengths and limitations

A major strength of the present study is the comprehensive characterization of parasitological, serological, immunological, and metabolic measurements in adults with metabolic disorders. This multidomain dataset allowed exploratory assessment of relationships between immune and metabolic measurements and infection status. The use of multiplex cytokine profiling together with logistic regression and ROC-based modeling enabled exploratory evaluation of biomarker combinations and their discriminatory performance. Another important strength is the evaluation of patients both before and after antiparasitic treatment, which allowed assessment of changes in immunological and metabolic measurements following treatment.

An important limitation is the final analytical sample of 80 participants. The study was prospectively designed for between-group comparisons and was not specifically designed for development of a multivariable prediction model. Accordingly, no model-specific sample-size calculation was performed before the study, and the multivariable model emerged as an exploratory analysis based on associations observed in the available data. A retrospective assessment based on the Riley framework and sensitivity scenarios for anticipated model performance and predictor complexity indicated that substantially larger cohorts would be required for stable prediction-model development (42). These calculations should not be interpreted as *post-hoc* power estimates, but they further support the interpretation of the present model as exploratory rather than as a validated diagnostic or predictive tool.

The data-driven variable-selection procedure may result in instability of the selected predictor combination and optimistic estimates of model performance. Although bootstrap resampling and 10-fold cross-validation were used to assess internal model performance, these procedures do not establish external validity. The additional sensitivity analysis showed that predictor-selection stability differed between variables, with IgM and BMI selected most frequently, whereas glucose, RANTES, and IL-12 were selected less consistently. Thus, the high internal discrimination of the integrated model should not be interpreted as evidence that all individual predictors are equally stable or that the selected combination is definitive. Independent validation in substantially larger and clinically diverse cohorts with prespecified predictors is required to determine whether the observed predictor combination and model performance are reproducible and clinically useful.

Finally, the observational design and the use of serum biomarkers limit the ability to establish temporal or causal relationships between metabolic and immunological alterations and active *G. intestinalis* infection. Although the study included pre- and post-treatment assessments, changes observed after treatment cannot be attributed solely to parasite eradication. The findings should therefore be regarded as hypothesis-generating and require confirmation in larger, prospective studies.

### 4.6 Future directions

Future studies should first confirm the observed immunological and metabolic associations in larger independent cohorts of adults with metabolic disorders and active *G. intestinalis* infection. Particular attention should be given to whether the observed differences in IL-12, IL-8, MCP-1, RANTES, HGF, immunoglobulins, and metabolic parameters are reproducible across populations and study settings.

If the exploratory predictor combination remains informative in independent cohorts, subsequent studies should prospectively prespecify candidate predictors and evaluate model performance in an adequately sized development cohort followed by independent external validation. Such studies should also determine whether the identified variables provide information beyond conventional parasitological and antigen-based diagnostic approaches.

Further prospective studies may additionally assess whether changes in immunological and metabolic measurements after antiparasitic treatment are reproducible and whether they have clinically meaningful relationships with treatment response. These questions require longitudinal designs specifically addressing treatment-related changes and should not be inferred from the present observational data.

## 5 Conclusions

The present study identified distinct immunological and metabolic patterns associated with active *Giardia intestinalis* infection in adults with metabolic disorders. The observed associations support further investigation of the interplay between immune and metabolic measurements in this clinical population. An exploratory multivariable analysis identified a combination of immunological and metabolic variables with promising discriminatory performance in the present cohort. However, this analysis was not prespecified as a prediction-model development study, and the available sample was not designed or sized for establishing a stable predictive model. The identified predictor combination should therefore be considered hypothesis-generating and requires evaluation in substantially larger, independent cohorts with prospective model development and external validation before its predictive or clinical utility can be determined.

## Data Availability

The original contributions presented in the study are included in the article/**Supplementary Material**. Further inquiries can be directed to the corresponding author.
