# Supplementary material for: Immunological and metabolic profiles associated with active Giardia intestinalis infection in adults with metabolic disorders: an exploratory analysis
Source: Front Immunol. 2026 Oct 27;17:1936690. doi: 10.3389/fimmu.2026.1936690 (PMC13747831; doi:10.3389/fimmu.2026.1936690)
Supplement: Supplementary file 1 [file Table1.docx]

**Supplementary Tables**

Table S1. Internal bootstrap validation of the final multivariable logistic regression model. The final analytical dataset comprised 80 participants, including 40 control participants and 40 participants with active *G. intestinalis* infection. The final model included glucose, BMI, IgM, RANTES, IL-12, and IL-2R. Bootstrap validation was based on 10,000 resamples generated by random sampling with replacement. Apparent AUC represents the discrimination of the model in the original analytical dataset. The 95% bootstrap percentile CI corresponds to the 2.5th and 97.5th percentiles of the bootstrap AUC distribution.

| **Measure** | **Result** |
| --- | --- |
| Final analytical dataset | 80 participants |
| Control group | 40 participants |
| Giardia-positive group | 40 participants |
| Bootstrap resamples | 10,000 |
| Final model predictors | Glucose, BMI, IgM, RANTES, IL-12, IL-2R |
| Apparent AUC | 0.953 |
| Mean bootstrap AUC | 0.953 |
| Median bootstrap AUC | 0.955 |
| 95% bootstrap percentile CI | 0.908–0.984 |

Abbreviations: AUC, area under the receiver operating characteristic curve; BMI, body mass index; CI, confidence interval; IgM, immunoglobulin M; IL-12, interleukin-12p40; IL-2R, interleukin-2 receptor; RANTES, regulated upon activation, normal T-cell expressed and secreted.

Note: The bootstrap analysis was performed using 10,000 samples generated by random sampling with replacement from the final analytical dataset. The 95% CI represents the 2.5th and 97.5th percentiles of the bootstrap AUC distribution.

Table S2. Internal performance of the final multivariable logistic regression model assessed using stratified 10-fold cross-validation, including the number of participants and folds, area under the receiver operating characteristic curve (AUC), accuracy, sensitivity, specificity, and numbers of true negatives (TN), false positives (FP), false negatives (FN), and true positives (TP).

| **Performance measure** | **10-fold cross-validation** |
| --- | --- |
| Number of participants | 80 |
| Number of folds | 10 |
| AUC | 0.954 |
| Accuracy | 85.0% |
| Sensitivity | 82.5% |
| Specificity | 87.5% |
| True negatives (TN) | 35 |
| False positives (FP) | 5 |
| False negatives (FN) | 7 |
| True positives (TP) | 33 |

Table S3. Predictor-selection stability in bootstrap sensitivity analysis

| **Predictor** | **Selection frequency** |
| --- | --- |
| IgM | 97.1% |
| BMI | 88.9% |
| IL-2R | 60.3% |
| IL-12 | 30.9% |
| RANTES | 29.3% |
| Glucose | 22.1% |

Selection frequencies represent the proportion of 10,000 bootstrap sensitivity-analysis samples in which the respective predictor was retained by the specified variable-selection procedure. This analysis was performed retrospectively as a sensitivity analysis and was not part of the original model-development procedure.
